# Carotid artery stenting with dual-layer stent implantation in a patient with complex anatomy

**DOI:** 10.1590/1677-5449.202501192

**Published:** 2026-08-28

**Authors:** Gislaine dos Santos Rodrigues Vieira, Ana Luiza Feitosa Leite de Souza, Crislayne dos Santos Rodrigues, João da Silva, Nelson Ogliari Rezende, William Wakasugui, Helen Caroline Magalhães Costa, Vinicius Tadeu Ramos da Silva Grillo

**Affiliations:** 1 Centro Universitário São Lucas – UNISL, Porto Velho, RO, Brasil.; 2 Instituto Vascular e Endovascular de Rondônia – IVER, Porto Velho, RO, Brasil.

**Keywords:** carotid stenosis, percutaneous transluminal angioplasty, anatomical variation

## Abstract

Carotid atherosclerotic disease associated with hemodynamically significant stenosis is a serious condition that requires accurate diagnosis and effective treatment to prevent cardiovascular events such as stroke. Treatment strategies, including carotid angioplasty and carotid endarterectomy, have evolved considerably, providing safer and less invasive therapeutic options. This report describes the case of a male patient with dyslipidemia, hypertension, and coronary artery disease who presented with near-occlusion of the left internal carotid artery caused by a plaque with features of instability. Given the lesion’s high embolic potential and the risk of embolization associated with conventional endovascular treatment, a dual-layer micromesh-covered CGuard^TM^ stent was used in conjunction with a distal embolic protection device. Despite the technical challenges related to the patient's vascular anatomy, the procedure was successfully completed, and the patient experienced a favorable clinical outcome, demonstrating the applicability of this technology in anatomically complex scenarios.

## INTRODUCTION

Extracranial carotid artery atherosclerotic disease is one of the main causes of ischemic stroke, accounting for approximately 20% of cases worldwide.^1,2^ Traditionally, carotid endarterectomy has been considered the gold-standard treatment for carotid artery stenosis, with its efficacy in preventing stroke demonstrated by multicenter randomized trials such as the North American Symptomatic Carotid Endarterectomy Trial and the European Carotid Surgery Trial.^1,3^ With advances in endovascular techniques and devices, carotid artery stenting has emerged as a viable alternative, particularly for patients considered at high risk for carotid endarterectomy.^2^

Despite their favorable results, first-generation stents, such as open-cell devices, has been associated with a higher risk of periprocedural neurological events due to plaque prolapse and embolization of debris through the stent cells.^2,4^ To overcome this limitation, dual-layer stents were developed.^4,5^ The CGuard^TM^ system (InspireMD, Tel Aviv, Israel), in particular, combines an open-cell nitinol stent with an external polyethylene terephthalate (MicroNet) mesh designed to trap embolic particles as small as 150-180 μm.^4-6^ Recent studies and meta-analyses involving low-risk populations have confirmed the safety and clinical efficacy of the CGuard^TM^ system, showing low rates of neurological complications and restenosis at 12 months.^7,8^

Endovascular treatment of patients with complex carotid anatomy, including severe vessel tortuosity and a high carotid bifurcation, presents a significant technical challenge.^9^ However, the flexibility and conformability of newer-generation stents, such as the CGuard^TM^, make them particularly well suited for these anatomies, allowing them to conform to the vessel without the risk of vessel straightening or technical failure observed with other devices.^6,10^

This case report describes the first carotid artery stenting procedure using the dual-layer CGuard^TM^ stent in a patient with complex carotid anatomy, representing a pioneering intervention in Northern Brazil. The aim is to detail the technical approach and discuss the immediate and short-term outcomes, thereby contributing real-world evidence to the literature on the use of this technology in challenging clinical scenarios. This study was reviewed and approved by a Research Ethics Committee (CAAE No. 78811724.3.0000.5297; approval No. 6.927.317).

### Part I – Clinical presentation

A 78-year-old male patient was followed in the outpatient setting for episodes of presyncope and syncope. His medical history was significant for hypertension, dyslipidemia, and coronary artery disease, including a previous myocardial infarction treated with percutaneous coronary intervention. Physical examination revealed no neurological deficits.

**Figure 1:**
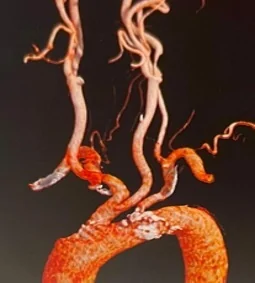
Computed tomography angiography with three-dimensional reconstruction of the aortic arch and supra-aortic vessels demonstrating luminal narrowing (critical stenosis) of the left internal carotid artery.

Color Doppler ultrasonography of the carotid and vertebral arteries revealed critical stenosis (> 70%) of the left internal carotid artery (ICA). Computed tomography angiography (CTA) of the carotid arteries confirmed diffuse atherosclerotic disease involving the aortic arch and carotid bifurcation, with a lipid-rich hypoechoic plaque causing near-occlusive stenosis of the left ICA. Cerebral CTA showed no evidence of acute or chronic ischemic lesions. The patient had a complex vascular anatomy, including a type II aortic arch and a left common carotid artery originating from the brachiocephalic trunk, an anatomical variation known as a bovine arch (Figure 1).

### Part II – What was done

In view of the presence of a lipid-rich near-occlusive plaque, complex vascular anatomy, and the patient’s high cardiovascular risk, endovascular treatment with carotid artery stenting was selected, using a dual-layer micro-mesh covered stent (CGuard™) to minimize the risk of periprocedural embolization. The procedure incorporated a distal embolic protection device (filter) to enhance procedural safety. According to national and international guidelines, the use of cerebral protection devices during carotid artery stenting is recommended and generally, except in the presence of specific technical contraindications, because it reduces the risk of periprocedural cerebral embolization.^1,2,11^

Alternatives treatment strategies, such as carotid endarterectomy sob under local anesthesia, could be considered, particularly in symptomatic patients with favorable anatomy.^1,3^ Another option would be TransCarotid Artery Revascularization, which is not yet available at our institution but may offer a lower risk of cerebral embolization in patients with challenging anatomy.^1,3^

The procedure was initiated with ultrasound-guided retrograde puncture of the right common femoral artery and placement of a 6 Fr x 11 cm introducer sheath, followed by intravenous bolus administration of unfractionated heparin (100 UI/kg)S. Selective catheterization of the left common carotid artery was attempted using a Simmons 2 catheter and a 0.035-inch guidewire. However, despite successful initial catheterization, the devices could not be safely advanced into the external carotid artery to allow exchange for a higher-support super-stiff guidewire.

The main technical challenge was achieving stable selective catheterization of the left external carotid artery to provide adequate support and allow advancement of the long sheath into the left common carotid artery. This limitation was attributable to the combination of a type II aortic arch and a bovine arch configuration, resulting in unfavorable angulation, system instability, and recurrent loss of support.

An alternative strategy was therefore adopted, consisting of ultrasound-guided puncture of the right brachial artery and placement of a 6 Fr introducer sheath. Through this access, selective catheterization of the left common and external carotid arteries was achieved using a 0.035-inch stiff guidewire supported by a 5 Fr vertebral catheter. After adequate distal anchoring, the wire was exchanged for a 0.035-inch super-stiff guidewire, allowing the advancement and positioning of a 7 Fr x 90 cm long sheath (Cook Medical) within the left common carotid artery.

**Figure 2:**
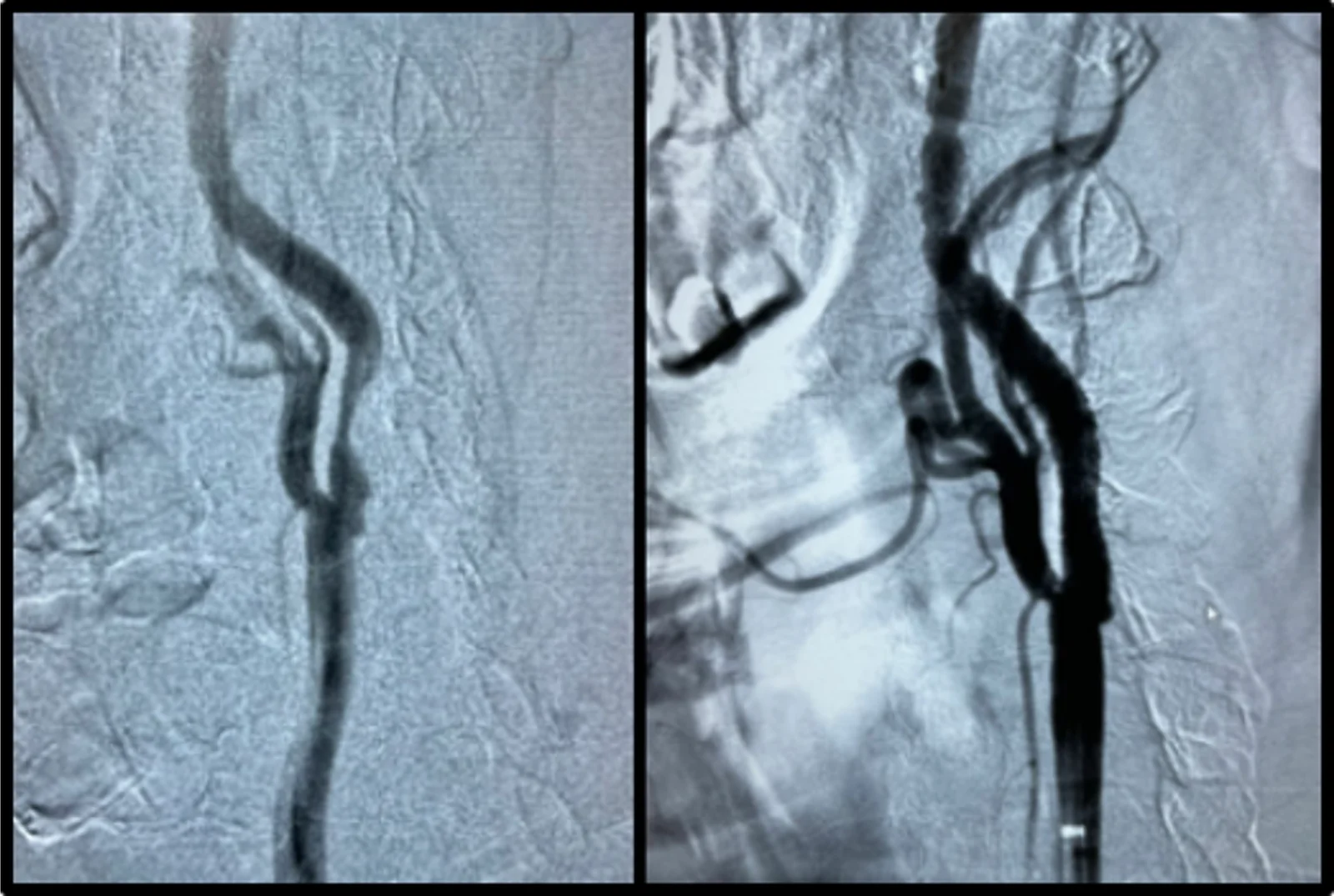
Digital subtraction angiography of the left carotid artery. Left: critical stenosis of the internal carotid artery. Right: completion angiography after carotid angioplasty and stent implantation*.*

After placement of the EZ™ distal embolic protection device (Boston Scientific, Natick, USA) in the ICA, an 8 x 40 mm CGuard™ stent (InspireMD, Tel Aviv, Israel) was deployed in the left ICA, followed by post-dilation with a 6 x 20 mm ballon to optimize stent apposition to the vessel wall. Completion angiography demonstrated complete resolution of the stenotic lesion, improved cerebral perfusion, and no evidence of embolic complications (Figure 2).

**Figure 3:**
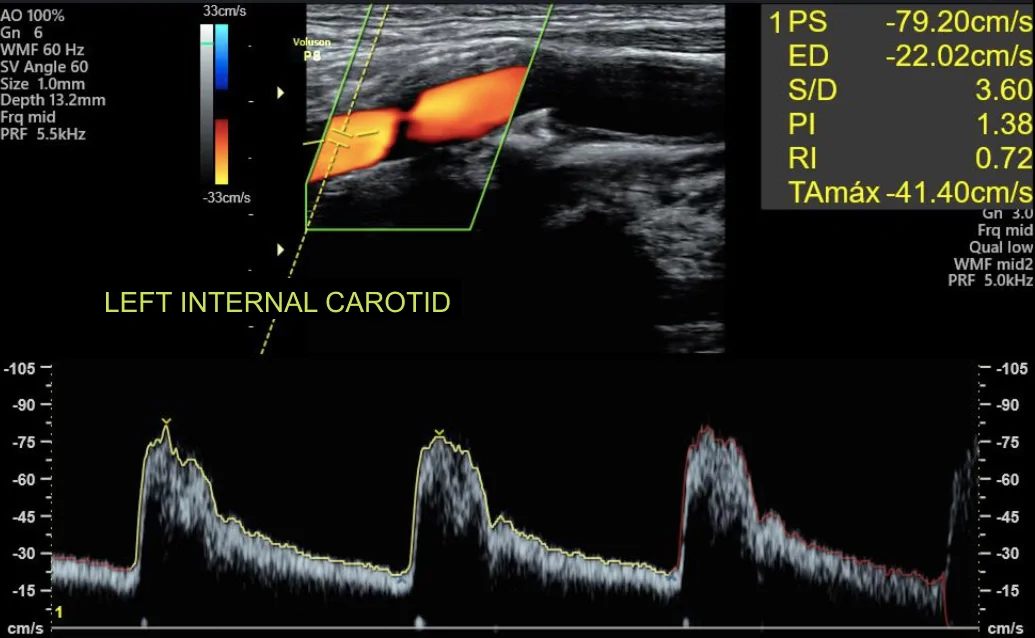
Doppler ultrasound of the left internal carotid artery performed 3 years after the procedure, demonstrating preserved patency of the previously implanted stent without evidence of significant stenosis. Spectral Doppler analysis showed systolic and diastolic velocities within normal limits for the treated segment, consistent with preserved hemodynamic function.

Postoperatively, the patient remained on dual antiplatelet therapy with aspirin 100 mg and clopidogrel 75 mg and experienced an uneventful clinical course. Follow-up Doppler ultrasound at 30 days demonstrated stent patency without evidence of restenosis. At late follow-up, performed 3 years after the procedure, the patient remained neurologically asymptomatic, and Doppler ultrasound confirmed continued stent patency with no signs of restenosis (Figure 3).

## DISCUSSION

The present case demonstrates the feasibility and success of carotid artery stenting with the dual-layer CGuard^TM^ stent in a patient with complex vascular anatomy. No neurological complications were observed during the periprocedural period or throughout clinical follow-up. These findings are consistent with recent evidence on dual layer stents, which has reported a 30-day stroke rate of 1.4% among patients undergoing carotid artery stenting with this technology.^4,7^

Evidence from multicenter studies such as IRONGUARD 2 and recent meta-analyses involving non-emergent population further support the favorable clinical profile of the CGuard^TM^ stent.^4,7,8^ IRONGUARD 2, the largest prospective registry evaluating dual-layer stents to date, reported adverse neurological (stroke/AIT) and 1-year restenosis rates of 0.68 and 0.82%, respectively. These outcomes remained consistent across different clinical and anatomical patient subgroups.^7^ Comparable results were observed in a prospective cohort of 113 patients, where CGuard^TM^ implantation was associated with a single case of in-stent occlusion leading to stroke (0.8%) and no additional case of restenosis over 12 months of follow-up.^8^ The technical success and absence of adverse events observed in the present case are consistent with these findings and further illustrate the device’s performance in a real world clinical setting and in a particularly challenging case.

The primary factor supporting the use of CGuard^TM^ stent in the present case was the presence of a lipid-rich plaque, a well-established predictor of cerebrovascular events.^2,12^ The dual-layer design of the CGuard^TM^ was developed to provide a more effective barrier against plaque protrusion through the stent cells and, consequently, to reduce periprocedural embolization.^4-6^ Comparative studies employing intraoperative monitoring have shown that carotid artery stenting is associated with a higher incidence of microembolic signals than endarterectomy, even when cerebral protection devices are used, underscoring the importance of technologies such as the CGuard™ in mitigating this risk.^13^

Furthermore, the flexibility and conformability of open-cell stents such as the CGuard^TM^ are superior to those of closed-cell stents in tortuous vessels.^2,5,6^ In an *in vitro* study, Wissgott et al.^6^ demonstrated that the CGuard^TM^ structure adapts well to vascular models with pronounced curvature and changes in vessel diameter, without causing significant vessel straightening, a critical factor in avoiding complications in complex anatomies.

The success achieved in the present intervention further supports the applicability of this type of stent in anatomically challenging cases. These findings should be interpreted in the context of studies such as that of Wodarg et al.^14^, which reported a higher incidence of serious adverse events, including periprocedural stroke and death, with open-cell stents (10.3%) compared with closed-cell stents (6.0%). Importantly, although the CGuard^TM^ is based on an open-cell design, it incorporates an external protective mesh intended to mitigate this risk, combining the flexibility of open-cell stents with embolic protection comparable to that of closed-cell devices.

Thrombogenicity and acute occlusion, which were initially considered potential concerns with certain dual-layer stents, such as the Casper-RX in emergency settings, were not observed in the present case. The meta-analysis by Pini et al. reinforces the safety of this technology, reporting an acute occlusion rate of only 0.8%.^4^ More recent evidence involving the CGuard^TM^ has likewise demonstrated low rates of acute occlusion in extremely high-risk populations, including patients with ischemic stroke and tandem lesions (4.8 and 9%, respectively). These rates are comparable to, or lower than, those reported for single-layer stents and markedly lower than those described for other dual-layer stent platforms.^10,15^

With regard to periprocedural anticoagulation, there are no specific recommendations for heparin dose adjustment when dual-layer stents are used. Administration of unfractionated heparin at a dose of 70-100 UI/kg during the procedure remains standard practice according to current guidelines.^1,2^ Postprocedural management continues to rely on dual antiplatelet therapy (aspirin plus clopidogrel), typically for a period of 1 to 3 months, followed by antiplatelet monotherapy. To date, there is no robust evidence supporting the routine use of full-dose anticoagulation or direct oral anticoagulants specifically for the prevention of carotid stent thrombosis, and such strategies remain reserved for patients with other well-established clinical indications.

Long-term stent durability and the prevention of restenosis remain important considerations.^11^ Although long-term follow-up of our patient is still ongoing, the 1-year results from IRONGUARD 2 (0.82%) and other CGuard^TM^ series (ranging from 0.41 to 4.05%) are encouraging and suggest that restenosis is an uncommon event with this device.^7,8^ These findings contrast with the higher rates reported for some first-generation dual-layer stents and point to a potential advantage of the CGuard^TM^ design in terms of reduced neointimal hyperplasia, which may contribute to sustained long-term patency.^7,16^

Despite the relevance of this pioneering case to clinical practice in the region, we acknowledge inherent limitations of a single case report, and its findings cannot be generalized. Nevertheless, the documentation of successful interventions in complex anatomies contributes to the growing body of evidence supporting the adoption of new technologies in clinical practice.

## CONCLUSION

CGuard^TM^ stent implantation in this patient with complex vascular anatomy was technically feasible, resulting in procedural success and no neurological complications during 3 years of follow-up. Although no conclusions regarding safety or efficacy at the population level can be drawn from a single case, this report suggests that dual-layer mesh-covered stents may represent a viable option in anatomically challenging scenarios, supporting the technical feasibility of an endovascular approach in this setting.

## Data Availability

Compartilhamento de dados não se aplica a este artigo, pois nenhum dado foi gerado ou analisado.
