# Supplementary material for: Carotid artery stenting with dual-layer stent implantation in a patient with complex anatomy
Source: J Vasc Bras. 2026 Aug 28;25:e20250119. doi: 10.1590/1677-5449.202501192 (PMC13560620; doi:10.1590/1677-5449.202501192)
Supplement: Translation

# Translation: Angioplastia de carótida com implante de stent recoberto de dupla malha em paciente de anatomia complexa

**DOI:** 10.1590/1677-5449.202501191

**Authors:** Gislaine dos Santos Rodrigues Vieira, Ana Luiza Feitosa Leite de Souza, Crislayne dos Santos Rodrigues, João da Silva, Nelson Ogliari Rezende, William Wakasugui, Helen Caroline Magalhães Costa, Vinicius Tadeu Ramos da Silva Grillo

**Affiliations:** 1 Centro Universitário São Lucas – UNISL, Porto Velho, RO, Brasil.; 2 Instituto Vascular e Endovascular de Rondônia – IVER, Porto Velho, RO, Brasil.

**Keywords:** estenose carotídea, angioplastia transluminal percutânea, variação anatômica

## Abstract

A aterosclerose carotídea associada à estenose hemodinamicamente significativa é uma condição grave que requer diagnóstico preciso e tratamento eficaz para prevenir eventos cardiovasculares, como o acidente vascular cerebral. As estratégias terapêuticas, incluindo a angioplastia carotídea e a endarterectomia carotídea, têm evoluído continuamente, proporcionando opções mais seguras e minimamente invasivas. O presente relato descreve o caso de um paciente do sexo masculino, portador de dislipidemia, hipertensão e coronariopatia, com diagnóstico de suboclusão da artéria carótida interna esquerda secundária a placa com características de instabilidade. Considerando o elevado potencial embólico da lesão e o risco de embolização associado ao tratamento endovascular convencional, optou-se pela utilização de um stent CGuard^TM^ recoberto por dupla malha micropermeável e sistema de proteção embólica distal. Apesar das dificuldades técnicas relacionadas às variações anatômicas do paciente, o procedimento foi realizado com sucesso, e a evolução clínica foi favorável, demonstrando a aplicabilidade dessa tecnologia em cenários anatômicos complexos.

## INTRODUÇÃO

A doença aterosclerótica da artéria carótida extracraniana é uma das principais causas de acidente vascular cerebral (AVC) isquêmico, sendo responsável por aproximadamente 20% dos casos em todo o mundo^1,2^. Tradicionalmente, a endarterectomia carotídea tem sido considerada o tratamento padrão-ouro para a estenose carotídea, com sua eficácia na prevenção de AVC comprovada por estudos multicêntricos randomizados, como o *North American Symptomatic Carotid Endarterectomy Trial* e o *European Carotid Surgery Trial* ^1,3^. Com os avanços nas técnicas e nos dispositivos endovasculares, a angioplastia com stent na artéria carótida emergiu como uma alternativa viável, especialmente para pacientes considerados de alto risco para a endarterectomia carotídea^2^.

Apesar dos resultados favoráveis, os stents de primeira geração, como os de célula aberta, foram associados a um maior risco de eventos neurológicos periprocedimento devido à protrusão de placa e à embolização de detritos através das células do stent^2,4^. Para contornar essa limitação, foram desenvolvidos os stents de dupla malha^4,5^. O sistema CGuard^TM^ (InspireMD, Tel Aviv, Israel), em particular, combina uma estrutura de nitinol de célula aberta com uma malha externa de tereftalato de polietileno (MicroNet), projetada para reter êmbolos de até 150-180 μm^4-6^. Estudos e metanálises recentes envolvendo populações de baixo risco têm corroborado a segurança e a eficácia clínica do CGuard^TM^, demonstrando baixas taxas de complicações neurológicas e de reestenose em 12 meses^7,8^.

O tratamento endovascular de pacientes com anatomia carotídea complexa, como tortuosidade acentuada e bifurcação carotídea alta, representa um desafio técnico significativo^9^. Contudo, a flexibilidade e a capacidade de adaptação dos stents de nova geração, como o CGuard^TM^, tornam esses dispositivos particularmente adequados para tais casos, permitindo sua conformação ao vaso sem o risco de retificação vascular ou falha técnica, como observado com outros dispositivos^6,10^.

Este relato descreve o primeiro caso de angioplastia de carótida com implante de stent de dupla malha CGuard^TM^ em um paciente com anatomia carotídea complexa, representando um procedimento pioneiro na região Norte do Brasil. O objetivo é detalhar a abordagem técnica empregada e discutir os resultados imediatos e de curto prazo, contribuindo com evidências do mundo real para a literatura sobre a utilização dessa tecnologia em cenários desafiadores. Este estudo foi devidamente avaliado e aprovado por um Comitê de Ética em Pesquisa (CAAE nº 78811724.3.0000.5297; parecer nº 6.927.317).

### Parte I – Situação clínica

Paciente do sexo masculino, 78 anos, em acompanhamento ambulatorial por episódios de lipotimia e síncope . Apresentava antecedentes de hipertensão arterial, dislipidemia e doença arterial coronariana (infarto agudo do miocárdio prévio tratado com angioplastia coronariana). Ao exame físico, não foram observados déficits neurológicos.

A ultrassonografia com Doppler colorido das artérias carótidas e vertebrais revelou estenose crítica (> 70%) da artéria carótida interna (ACI) esquerda. A angiotomografia das artérias carótidas confirmou a presença de aterosclerose difusa no arco aórtico e na bifurcação carotídea, com uma placa hipoecogênica rica em lipídios na ACI esquerda, de padrão suboclusivo. A angiotomografia cerebral não evidenciou lesões isquêmicas agudas ou crônicas. A anatomia vascular do paciente era complexa, com arco aórtico tipo II e origem da artéria carótida comum esquerda a partir do tronco braquiocefálico, uma variação anatômica conhecida como arco bovino (Figura 1).

**Figura 1:** Angiotomografia com reconstrução tridimensional do arco aórtico e dos troncos supra-aóritcos, evidenciando redução luminal (estenose crítica) da artéria carótida interna esquerda.

### Parte II – O que foi feito

Considerando a presença de uma placa suboclusiva rica em lipídios, a anatomia vascular complexa e o elevado risco cardiovascular do paciente, optou-se pelo tratamento endovascular por meio de angioplastia carotídea, utilizando-se um stent recoberto de dupla malha micropermeável (CGuard™) com o objetivo de reduzir o risco de embolização periprocedimento. A intervenção foi realizada com sistema de proteção embólica distal (filtro), visando maximizar a segurança. Ressalta-se que, de acordo com diretrizes nacionais e internacionais, o uso de dispositivos de proteção cerebral durante a angioplastia carotídea é recomendado e considerado mandatório, exceto em situações de contraindicação técnica específica, por reduzir o risco de embolização cerebral periprocedimento^1,2,11^.

Alternativas como a endarterectomia carotídea sob anestesia local poderiam ser consideradas, especialmente em pacientes sintomáticos e com anatomia favorável^1,3^. Outra possibilidade seria a revascularização transcarotídea (*TransCarotid Artery Revascularization*), ainda indisponível em nosso serviço, mas que poderia oferecer menor risco embólico em pacientes com anatomia desafiadora^1,3^.

O procedimento foi iniciado com punção retrógrada ecoguiada da artéria femoral comum direita e implante de um introdutor 6 Fr x 11 cm, seguido da administração intravenosa em *bolus* de heparina não fracionada na dose de 100 UI/kg. Realizou-se tentativa de cateterização seletiva da artéria carótida comum esquerda utilizando cateter Simmons 2 e fio-guia de 0,035”. Entretanto, apesar da cateterização seletiva inicial, não foi possível avançar os dispositivos com segurança até a artéria carótida externa para realizar a troca por um fio-guia de maior suporte (*super stiff*).

A dificuldade técnica consistiu na obtenção de um cateterismo seletivo estável da artéria carótida externa esquerda, necessário para proporcionar suporte adequado e permitir o avanço da bainha longa até a artéria carótida comum esquerda. Essa limitação decorreu da presença de arco aórtico tipo II associado à variação anatômica de arco bovino, resultando em angulação desfavorável, instabilidade do sistema e perda recorrente de suporte.

Optou-se, então, por um acesso alternativo, com punção ecoguiada da artéria braquial direita e implante de um introdutor 6 Fr. Por esse acesso, realizou-se a cateterização seletiva das artérias carótida comum e externa esquerdas utilizando fio-guia *stiff* de 0,035” sob suporte de cateter vertebral 5 Fr. Após adequada ancoragem distal, procedeu-se à troca para um fio-guia *super stiff* de 0,035”, permitindo o avanço e o posicionamento de uma bainha longa 7 Fr x 90 cm (Cook Medical) na artéria carótida comum esquerda.

Após o posicionamento do filtro de proteção embólica distal EZ™ (Boston Scientific, Natick, EUA) na ACI, foi realizado o implante de um stent CGuard™ 8 x 40 mm (InspireMD, Tel Aviv, Israel) na ACI esquerda, seguido de pós-dilatação com balão de 6 x 20 mm para otimizar a aposição do stent à parede vascular. A angiografia de controle demonstrou correção completa da estenose, com melhora da perfusão cerebral e ausência de complicações embólicas (Figura 2).

**Figura 2:** Angiografia por subtração digital da artéria carótida esquerda. Esquerda: observa-se estenose crítica da artéria carótida interna. Direita: angiografia de controle após angioplastia com implante de stent*.*

No pós-operatório, o paciente manteve terapia antiplaquetária dupla com aspirina 100 mg e clopidogrel 75 mg, evoluindo sem intercorrências clínicas. O exame de ultrassonografia Doppler de controle aos 30 dias demonstrou perviedade do stent, sem evidências de reestenose. Em reavaliação tardia, realizada 3 anos após o procedimento, o paciente permanecia neurologicamente assintomático, e o Doppler evidenciou plena perviedade do stent previamente implantado, sem sinais de reestenose (Figura 3).

**Figura 3:** Ultrassonografia vascular com Doppler da artéria carótida interna esquerda realizada 3 anos após o procedimento, demonstrando perviedade preservada do stent previamente implantado, sem sinais de reestenose significativa. A análise espectral evidenciou velocidades sistólica e diastólica dentro dos limites da normalidade para o segmento tratado, indicando manutenção da adequada função hemodinâmica.

## DISCUSSÃO

O presente caso demonstra a viabilidade e o sucesso da angioplastia de carótida com o stent de dupla malha CGuard^TM^ em um paciente com anatomia vascular complexa. Não foram observadas complicações neurológicas durante o período periprocedimento nem durante o seguimento clínico. Esse achado está em consonância com a literatura recente sobre stents de dupla malha, que relata taxa de AVC em 30 dias de 1,4% em pacientes submetidos a angioplastia carotídea com essa tecnologia^4,7^.

Estudos multicêntricos como o IRONGUARD 2, e metanálises recentes em populações não emergenciais reforçam o perfil favorável do CGuard^TM4,7,8^. O IRONGUARD 2, o maior registro prospectivo sobre o uso de stents de dupla malha até o momento, relatou taxa de eventos neurológicos adversos (AVC/AIT) de 0,68% e taxa de reestenose de 0,82% em 1 ano, resultados que permaneceram consistentes independentemente das características clínicas e anatômicas dos pacientes^7^. Resultados semelhantes foram observados em estudo prospectivo com 113 pacientes, no qual o uso do CGuard^TM^ esteve associado a apenas um evento de oclusão intra-stent com AVC (0,8%) e à ausência de outros casos de reestenose durante 12 meses de seguimento^8^. Nossos achados, incluindo o sucesso técnico e a ausência de eventos adversos, refletem o desempenho do dispositivo em um cenário de mundo real e em um caso particularmente desafiador.

O principal fator que justificou a escolha do CGuard^TM^ no presente caso foi a presença de uma placa rica em lipídios, considerada um importante preditor de eventos cerebrovasculares^2,12^. A tecnologia de dupla malha do CGuard^TM^ foi desenvolvida para proporcionar uma barreira mais eficaz contra a protrusão de material da placa através das células do stent e, consequentemente, reduzir a embolização periprocedimento^4–6^ . Estudos comparativos com monitorização intraoperatória demonstraram que a angioplastia carotídea está associada a maior incidência de sinais de microembolização quando comparada à endarterectomia carotídea, mesmo com o uso de dispositivos de proteção cerebral, reforçando a relevância de tecnologias como o CGuard™ para mitigar esse risco^13^.

Além disso, a flexibilidade e a capacidade de adaptação dos stents de célula aberta, como o CGuard^TM^, são superiores às dos stents de célula fechada em vasos tortuosos^2,5,6^. Em estudo experimental *in vitro*, Wissgott et al.^6^ demonstraram que a estrutura do CGuard^TM^ se adapta adequadamente a modelos vasculares com curvas acentuadas e variações de diâmetro, sem promover retificação significativa do vaso, um aspecto fundamental para evitar complicações em anatomias complexas.

O sucesso da presente intervenção reforça a aplicabilidade desse tipo de stent em casos anatomia desafiadora. Esse achado deve ser interpretado à luz de estudos como o de Wodarg et al.^14^ que demonstraram maior risco de eventos adversos graves, incluindo AVC periprocedimento e óbito, com stents de célula aberta (10,3%) em comparação com stents de célula fechada (6,0%). Entretanto, é importante destacar que o CGuard^TM^, embora possua uma estrutura de célula aberta, incorpora uma malha externa de proteção destinada a mitigar esse risco, combinando a flexibilidade dos stents de célula aberta com um nível de proteção embólica semelhante ao dos stents de célula fechada.

A trombogenicidade e a oclusão aguda, inicialmente consideradas potenciais limitações de alguns stents de dupla malha, como o Casper-RX em cenários de emergência, não foram observadas neste caso. A metanálise de Pini et al.^4^ reforça a segurança dessa tecnologia, demonstrando taxa de oclusão aguda de apenas 0,8% para stents de dupla camada. Estudos mais recentes com o CGuard^TM^ também têm relatado baixas taxas de oclusão aguda em populações de altíssimo risco em pacientes de AVC agudo e lesões tandem (4,8 e 9%, respectivamente), resultados comparáveis ou até inferiores aos observados com stents de camada única e substancialmente inferiores aos relatadas para outros stents de dupla malha^10,15^.

No que se refere à anticoagulação periprocedimento, não há recomendações específicas para ajuste da dose de heparina em procedimentos com stents de dupla malha. A administração de heparina não fracionada na dose de 70-100 UI/kg durante o procedimento permanece a prática padrão descrita nas diretrizes^1,2^. Em relação ao tratamento após o procedimento, a terapia antiplaquetária dupla (aspirina associada ao clopidogrel) continua sendo o regime recomendado, geralmente por um período de 1 e 3 meses, seguida de monoterapia antiplaquetária. Até o momento, não há evidências robustas que justifiquem o uso rotineiro de anticoagulação plena ou de anticoagulantes orais diretos com o objetivo específico de prevenir trombose de stent carotídeo, permanecendo essa estratégia restrita a indicações clínicas concomitantes bem estabelecidas.

A durabilidade do stent e a prevenção de reestenose permanecem aspectos importantes a serem considerdos^11^. Embora o seguimento de longo prazo do nosso paciente ainda esteja em andamento, os resultados de 1 ano do estudo IRONGUARD 2 (taxa de reestenose de 0,82%) e de outras séries envolvendo o CGuard^TM^ (variando de 0,41 a 4,05%) são promissores e sugerem que a reestenose é um evento incomum com este dispositivo^7,8^. Esses achados contrastam com taxas mais elevadas descritas para alguns stents de dupla malha de primeira geração e sugerem uma possível vantagem do desenho do CGuard^TM^ em termos de menor hiperplasia neointimal, contribuindo para a manutenção da perviedade a longo prazo^7,16^.

Apesar da relevância deste caso pioneiro para a prática clínica na região, reconhecemos as limitações inerentes a um relato de caso único, cujos resultados não podem ser generalizados. No entanto, a documentação de intervenções bem-sucedidas em anatomias complexas contribui para o corpo de evidências que sustenta a incorporação de novas tecnologias à prática clínica.

## CONCLUSÃO

O implante do stent CGuard^TM^ neste paciente com anatomia complexa foi tecnicamente factível e resultou em sucesso do procedimento, sem ocorrência de complicações neurológicas durante os 3 anos de seguimento. Embora não permita inferências sobre segurança ou eficácia em nível populacional, este relato sugere que a utilização de stent recoberto de dupla malha pode representar uma alternativa viável em cenários anatômicos desafiadores, reforçando a factibilidade da estratégia endovascular nesse contexto.

## Data Availability

Data sharing is not applicable to this article as no new data were generated or analyzed.
